# Safety Evaluation in Healthy Adults of Motion-Based Virtual Reality Dichoptic Training for Pediatric Patients With Amblyopia: Prospective Intervention Study

**DOI:** 10.2196/69801

**Published:** 2025-06-17

**Authors:** Masakazu Hirota, Yuichi Okumura, Ken Nagino, Takao Hayashi, Takashi Negishi, Shintaro Nakao, Hitoshi Kawasaki, Takenori Inomata

**Affiliations:** 1 Department of Orthoptics, Faculty of Medical Technology, Teikyo University, Itabashi-ku, Tokyo, Japan; 2 Department of Ophthalmology, School of Medicine, Teikyo University, Itabashi-ku, Tokyo, Japan; 3 Graduate Degree Program of Health Data Science, Teikyo University, Itabashi-ku, Tokyo, Japan; 4 Graduate Degree Program of Comprehensive Applied Data Science, Teikyo University, Itabashi-ku, Tokyo, Japan; 5 InnoJin Inc, Bunkyo-ku, Tokyo, Japan; 6 Department of Ophthalmology, Juntendo University Graduate School of Medicine, 2-1-1 Hongo, Bunkyo-ku, Tokyo, 1130033, Japan, 81 338133111; 7 Department of Telemedicine and Mobile Health, Juntendo University Graduate School of Medicine, Bunkyo-ku, Tokyo, Japan; 8 Department of Hospital Administration, Juntendo University Graduate School of Medicine, Bunkyo-ku, Tokyo, Japan; 9 Ima-Create Inc, Shinagawa-ku, Tokyo, Japan; 10 Data Science, Juntendo University Graduate School of Medicine, Bunkyo-ku, Tokyo, Japan

**Keywords:** amblyopia, adherence, safety evaluation, virtual reality, dichoptic training, strabismus, digital health, digital therapeutics, visually induced motion sickness

## Abstract

**Background:**

Amblyopia, a unilateral or bilateral visual disorder, affects up to 5% of the general population and is a leading cause of childhood visual impairment. Current treatments, such as patching therapy, aim to improve amblyopia by temporarily occluding the unaffected eye, thereby promoting the use of the amblyopic eye. However, adherence to patch therapy can be challenging, as the forced use of the amblyopic eye can be stressful for children. Moreover, despite improvements in visual acuity by patch therapy, children with amblyopia often face difficulties with hand-eye coordination; therefore, a treatment that reduces stress for them while simultaneously improving hand-eye coordination could address the limitations of existing amblyopia therapies.

**Objective:**

This study investigated the safety of our motion-based virtual reality (VR) dichoptic training app using Japanese Kendama in healthy adult participants, which was designed to improve hand-eye coordination in pediatric patients with amblyopia.

**Methods:**

This prospective intervention study involved 20 healthy young adults (median age 21, IQR 21‐28.3 y), including 16 women. The participants played the motion-based VR dichoptic training app for 30 minutes and then completed a subjective symptom questionnaire, which comprised 9 questions (Q1-Q9) with each item scored on a 4-point scale, except Q9, which was assessed on a binary scale. Q1-Q3 focused on subjective eye symptoms, Q4-Q7 evaluated physical and mental discomfort, Q8 assessed the degree of VR session–induced arm fatigue, and Q9 assessed the severity of visually induced motion sickness.

**Results:**

No significant differences were observed in the reported ocular symptoms before and after the VR session, including eye fatigue (mean before vs after: 1.25, SD 0.94 points vs 1.35, SD 0.85 points), blurred vision (0.55, SD 0.50 points vs 0.80, SD 0.40 points), eye dryness (0.95, SD 0.74 points vs 1.25, SD 0.83 points), and visually induced motion sickness (0.00, SD 0.00 points vs 0.05, SD 0.22 points). These results suggested that the motion-based VR dichoptic training did not induce significant adverse ocular effects.

**Conclusions:**

The motion-based VR dichoptic training app demonstrated minimal adverse ocular effects in healthy adult participants, suggesting that it is safe for use in this population. These findings demonstrate the feasibility and good tolerability of this VR-based intervention in healthy adults. Further studies, including clinical studies in adult and pediatric patients with amblyopia, are warranted to evaluate its applicability and therapeutic effects.

## Introduction

Amblyopia, defined as a unilateral or bilateral visual disorder, affects up to 5% of the general population [1-3] and is a common cause of childhood visual impairment [4]. Refractive error correction with spectacles is the first-line treatment for pediatric amblyopia, regardless of the cause [5,6]. However, occlusion treatment (patching therapy), which involves blocking the unaffected eye to force the use of the amblyopic eye, may be required in some cases [7]. While combining corrective spectacles and patching therapy can improve visual acuity and shorten the treatment duration [8], treatment adherence and patient stress are significant clinical challenges. Studies have shown that treatment adherence to patching therapy is often <60% [9,10]. Many children with amblyopia dislike wearing eye patches, and there is a risk of vision impairment in the healthy eye with prolonged patching. Additionally, children with amblyopia treated with patching therapy often demonstrate lower binocular function than normal children [11]. By forcing the use of monocular vision during their developmental period, patching therapy may inhibit the development of normal binocular visual function in children.

Dichoptic training, which was presented by Hess et al [12], offers a promising approach to improving visual acuity in pediatric patients with amblyopia. This method involves the presentation of contrasting visual stimuli to each eye to restore binocular fusion, achieve stereopsis, and improve visual acuity in the amblyopic eye. Video games have emerged as a suitable platform for dichoptic training owing to their inherent engagement, allowing the promotion of binocular vision while treating amblyopia [13,14]. Furthermore, the contrast manipulation method in dichoptic training translates well to virtual reality (VR) head-mounted displays (HMDs) [15]. VR allows users to interact with and navigate a computer-generated environment in real time. Several studies have already explored the therapeutic effects of VR games for amblyopia treatment [16-18]. Žiak et al [16] reported a 30% improvement in visual acuity following 1 month of VR-HMD training (Vivid Vision Inc). Xiao et al [18] similarly showed that 12 weeks of VR-HMD dichoptic training, which used contrast manipulation and blacking out specific video regions (Luminopia Inc), improved the visual acuity of amblyopic eyes. These findings suggest that the outcomes of VR-based approaches are comparable to conventional patching therapy in treating amblyopia. By incorporating digital, game-like elements, VR-based approaches demonstrate comparable efficacy to patching therapy, potentially enhancing adherence and engagement [19,20].

**Figure 1.**
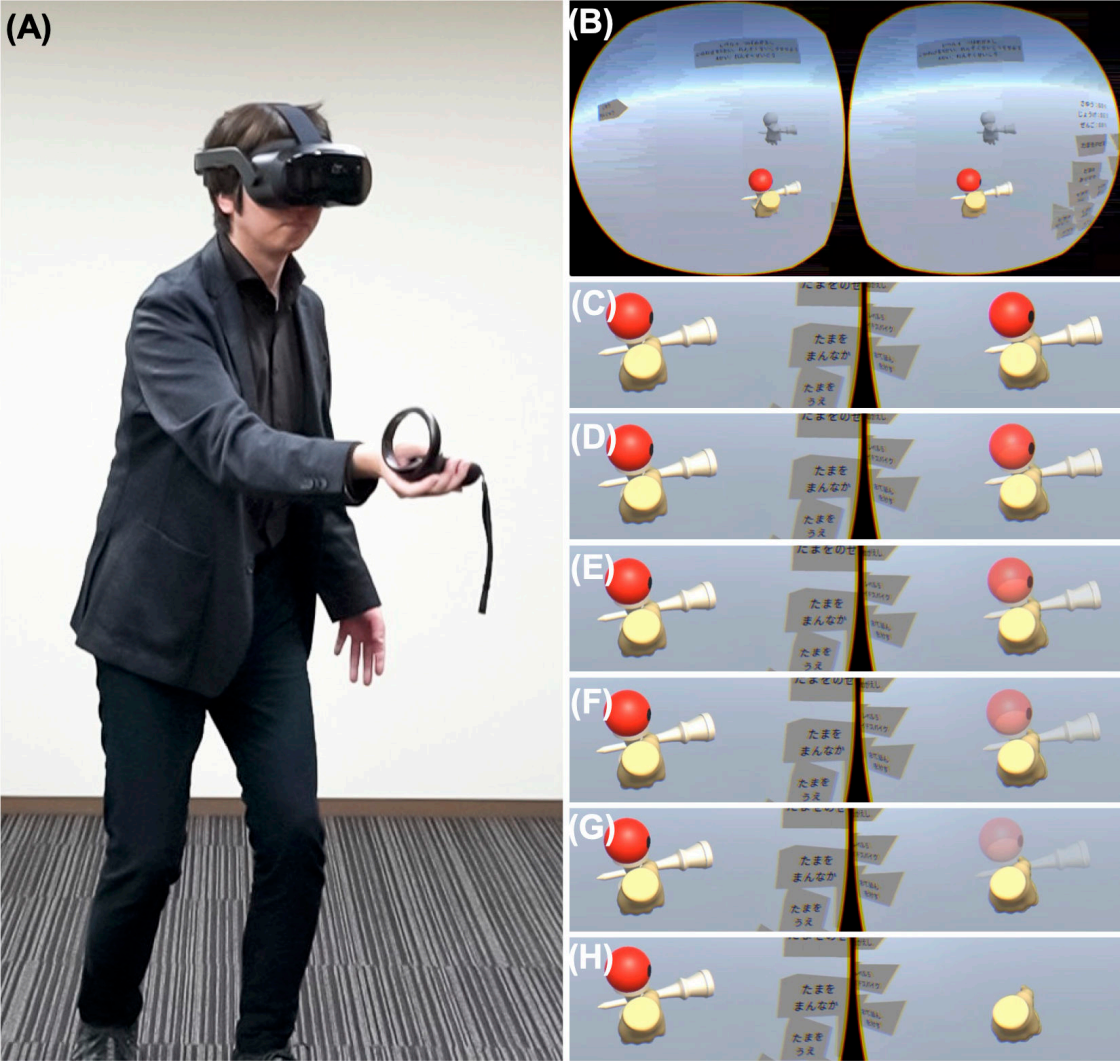
Images of the VR-based dichoptic training app. (A) The VR HMD is worn by the participant. The right-hand controller is linked to the ken (handle). (B) Screenshot of the VR-based dichoptic training app using Japanese Kendama, while watching the ideal Kendama movement in grayscale. (C-H) Transmittance of one eye (dominant eye) can be changed from 100% to 0% in 20% decrements (C: 100%; D: 80%; E: 60%; F: 40%; G: 20%; and H: 0%). HMD: head-mounted display; VR: virtual reality.

Previous studies on VR games for amblyopia primarily focused on nonengaging content, such as passive image viewing or using conventional consumer game controllers, which underuse the full potential of VR technology to enhance hand-eye coordination [16-18]. Children with amblyopia typically exhibit reduced hand-eye coordination [21,22]; however, its improvement with amblyopia treatment has not been widely established. To address this limitation, we developed a motion-based VR dichoptic training app (Figure 1) aimed at enhancing hand-eye coordination in pediatric patients with amblyopia. Unlike previous VR games for amblyopia that involve passive viewing, our app uses motion-tracking hand controllers that allow real-time manipulation of the virtual world, mirroring the user’s actual hand movements. This real-time movement may also lead to higher risks of visual fatigue and visually induced motion sickness (VIMS) in pediatric patients with amblyopia compared to previous VR treatments, which involve less active engagement. Additionally, the use of motion-based VR dichoptic training apps in clinical trials for pediatric patients with amblyopia necessitates resolving ethical considerations, such as safety and efficacy, due to the time-sensitive nature of amblyopia treatment.

In this study, we investigated the safety of our novel motion-based VR dichoptic training app in healthy young adults; in the future, the results of this study will be validated first in adult patients with amblyopia, and thereafter, in pediatric patients with amblyopia.

## Methods

### Participants

Participants for this preliminary study were recruited through posters displayed on the university campus of Teikyo University (Tokyo, Japan) and announcements on our website. A total of 20 healthy young adult volunteers were enrolled. All participants underwent comprehensive ophthalmologic examinations, including ocular dominance assessment using the hole-in-the-card test, best-corrected visual acuity evaluation at a distance (5 m), near the point of convergence measurement, stereoscopic acuity evaluation at 40 cm (Titmus Stereo test; Stereo Optical Co, Inc), heterophoria assessment using the alternating cover test at both near (33 cm) and far (5 m) distances, and fundus examination. Stereoacuity was converted to logarithm of arcsec (log arcsec) at Teikyo University. Participants were excluded if they exhibited any of the following characteristics: a best-corrected visual acuity of <20/20, a near point of convergence of >8 cm, stereoacuity of >100 arcsec, and the presence of manifest strabismus (including intermittent exotropia) or any retinal abnormalities.

### Ethical Considerations

Written informed consent was obtained from all participants after a thorough explanation of the study procedures and potential risks. This study was approved by the Institutional Review Board of Teikyo University (22–061) and was conducted in accordance with the principles of the Declaration of Helsinki. To ensure participant privacy and confidentiality, all collected data were anonymized and deidentified before analysis. Participants were informed that no compensation would be provided as the study involved minimal intervention. Additionally, no identifiable images of participants were included in the study or supplementary materials.

### Motion-Based VR Dichoptic Training App for Pediatric Amblyopia

**Figure 2.**
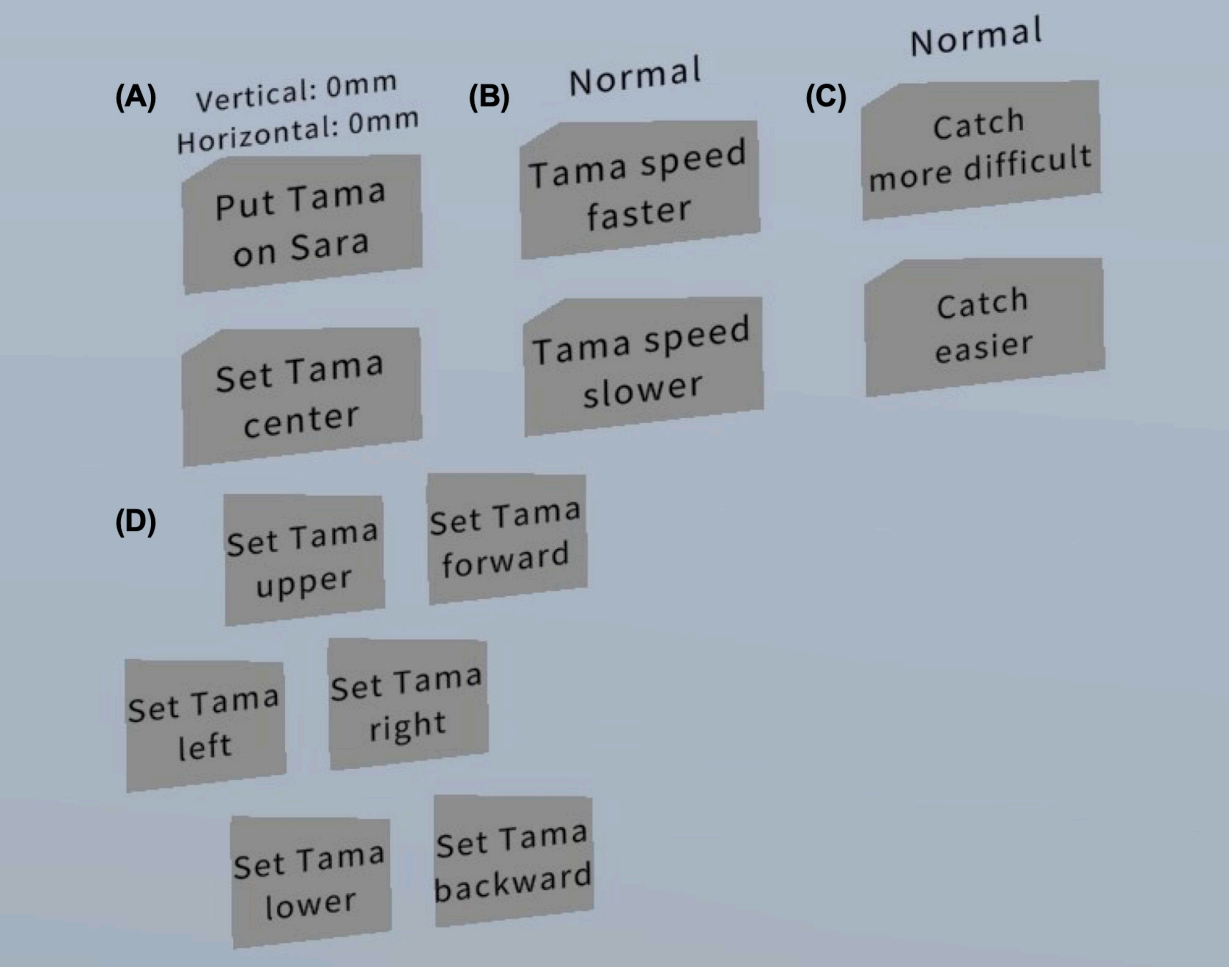
Screenshots of the setting panels for the VR-based dichoptic training app. (A) Restart function. If the waza fails and the tama falls to the ground, the participants touch “Put Tama on Sara.” (B) Speed adjustment function. The ball speed can be changed by touching “Tama speed faster” or “Tama speed slower” in 3 levels: “slow,” “normal,” and “fast.” (C) Difficulty adjustment function. When participants try to play waza, it can be determined whether the tama is regarded as a success even if it slightly deviates from the sara or whether it is played at the same difficulty level as real Kendama (“difficult”) by touching the “Catch more difficult” or “Catch easier” in 3 levels: “easy,” “normal,” and “difficult.” (D) Position alignment function. In VR, the binocular disparity may be larger than in real space because images are presented with a disparity between the left and right eyes. For some participants, the tama may not be aligned on the sara, so the offset of the balls is adjusted by touching the “Set Tama upper, lower, left, right, forward, and backward” panel. VR: virtual reality*.*

A motion-based VR dichoptic training app using Japanese Kendama was developed using VIVE Focus 3 (HTC Corp) on Unity (version 2020.3.13; Unity Technologies; Figure 1A). The controller for the right hand was linked to a ken (handle), as its weight (145 g) approximated the weight of the actual ken (140 g). Participants can adjust the physical and spatial parameters of the tama (ball) through an in-VR touch panel (Figure 2). The speed of the tama can be set to 1.0, 0.7, or 0.4 times that of the real-world speed. Difficulty levels for placing the tama on the Kendama are selectable as “easy,” “normal,” and “difficult,” with “difficult” simulating real-world Kendama difficulty. As the VR space presents parallax images to each eye, touch panels are available for the horizontal, vertical, forward, and backward adjustments of the tama’s position. An additional touch panel allows participants to restart the game by placing the tama on the sara (dishes) if it falls on the ground.

Figure 1B shows a screenshot of the motion-based VR dichoptic training app using Japanese Kendama while watching the ideal Kendama movement in grayscale. The examiner controlled the Kendama environment using a left-hand controller, which can adjust the tama’s transparency in one eye (Figure 1C-H; 100%, 80%, 60%, 40%, 20%, or 0%; completely transparent). Furthermore, it is possible to completely separate the visual stimuli for each eye, displaying the ken and tama correspondingly.

### Kendama Task

Participants initiated the Kendama task by selecting the “Challenge” panel on the touch screen. This task comprised 5 wazas (techniques): “Ozara (big dish),” “Kozara (small dish),” “Moshikame (a basic repetitive catching technique),” “Orbit (a trick where the ball moves in a circular path around the spike),” and “Side spike (a technique where the ball is caught on the side spike of the kendama).” Before each attempt, the system displayed an instructional video demonstrating the ideal Kendama movement of the waza being performed. The task was designed such that successful completion of a waza 5 consecutive times allowed progression to the next waza.

### Subjective Symptom Questionnaire

**Figure 3.**
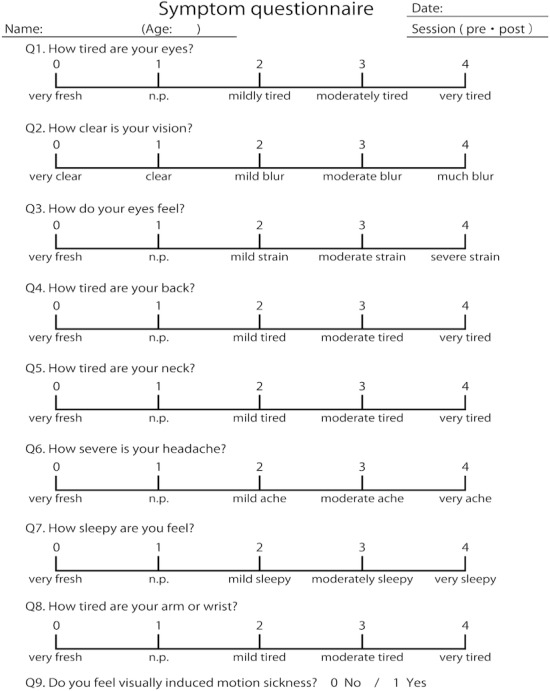
Subjective symptom questionnaire. Q1-Q3 assess subjective eye symptoms; Q4-Q7 assess physical and mental discomfort; Q8 evaluates the degree of VR session–induced arm fatigue; and Q9 evaluates VIMS. n.p.: no problem; VIMS: visually induced motion sickness; VR: virtual reality*.*

Participants were instructed to complete a subjective symptom questionnaire before and after the VR session. The questionnaire was adapted from the previous studies of Nakazawa et al [23], Sheedy and Bergstrom [24], and Hoffman et al [25]. Q1-Q7 were the same as those used in a previous study (Figure 3) [26-28]. Specifically, Q1-Q3 assessed subjective eye symptoms, and Q4-Q7 assessed physical and mental discomfort. Q1-Q7 have been widely used in previous studies to evaluate eye strain, as well as physical and mental discomfort associated with the use of digital devices. Q8 was designed to evaluate the degree of VR session-induced arm fatigue, and Q9 was designed to evaluate VIMS. Participants scored each question on a 4-point scale (0-4), except for Q9, which was scored as either 0=no or 1=yes point.

### Experimental Procedures

In this study, participants completed a subjective symptom questionnaire before and after the 30-minute motion-based VR dichoptic training sessions at Teikyo University. The duration was determined based on prior studies [27,29]. Participants’ refractive errors were corrected using soft contact lenses (SEED 1 day Pure moisture, Seed Co, Ltd) because VR-HMD is difficult to wear with glasses. With contact lenses, all participants had equal or better than 0.0 logMAR visual acuity. The Kendama task was performed at a tama speed of 0.4×, and the difficulty level was set to “easy.”

Initially, both eyes received a transmittance of 100%. Upon successful completion of the 5 wazas, the transmittance of the right eye (assumed to be healthy) was reduced to 60%, defined as the second condition. After completing all 5 wazas under the second condition, the transmittance of the right eye was reduced to 20%, which is defined as the third condition. Similarly, upon task completion under the third condition, participants performed the 5 wazas in the final condition, where only the tama and ken were displayed in the right and left eyes, respectively. The progression of transmittance reduction was designed as a game mechanic to gradually increase task difficulty and maintain participant engagement, rather than being a direct intervention for amblyopia treatment. The primary goal of this phase was to assess the feasibility and usability of the VR app with healthy participants rather than applying clinical treatment principles. The decision to reduce transmittance consecutively (and not simultaneously) was made to incrementally challenge the participants’ ability to complete the task while maintaining their attention and engagement. Notably, this procedure is distinct from contrast balancing used in clinical dichoptic training for amblyopia. In clinical settings, adjustments to transmittance would be customized based on the patient’s visual acuity and would likely involve simultaneous changes to both eyes, rather than the sequential reduction observed in this study. Additionally, contrast adjustments for amblyopia therapy would aim to improve visual function, whereas the transmittance changes in this study were primarily intended to serve as part of a gamified challenge.

### Statistical Analysis

This study investigated the continuous response variable from matched pairs of participants. A previous study indicated that the difference in responses between matched pairs was nonnormally distributed with an SD of 0.60 [27]. Assuming a true difference of 0.58 in the mean response of matched pairs, 18 pairs of participants would be required to be able to reject the null hypothesis (“The response difference is zero”) with a probability (power) of 0.8. The Type I error probability to test the null hypothesis was 0.05. Anticipating a 10% dropout rate owing to missing data or consent withdrawal, a total of 20 patients were needed.

Continuous variables are presented as median (IQR) or mean (SD). Differences in subjective symptom scores before and after motion-based VR dichoptic training sessions were analyzed using the Wilcoxon signed rank test. The *P* values for subjective symptoms were adjusted using Holm correction. Statistical significance was determined using SPSS Statistics for Windows (version 26; IBM Corp) with a significance level of *P*<.05.

## Results

### Characteristics of Participants

Ocular deviations with minus and plus signs indicate exodeviation and esodeviation, respectively.

The median age of the participants was 21 (21‐28.3) years, and 16 of 20 (80%) participants were women. The mean spherical equivalent refractive errors were −3.36 (SD 3.14) diopter (D) and −3.32 (SD 3.09) D for the dominant and nondominant eyes, respectively. All participants demonstrated a best-corrected visual acuity of 0.0 logMAR units or better. Mean heterophoria values were −0.4 (SD 0.8) prism diopter and −3.5 (SD 4.0) prism diopter at distance and near proximity, respectively. All healthy volunteers exhibited a stereoacuity of 1.60 log arcsec (40 s).

Table 1 presents the participant characteristics. This study included 20 healthy young adults.

**Table 1.** Characteristics of participants.

			Spherical equivalent (D)^a^			Ocular deviation (PD^b^)^c^	
ID	Age (years)	Sex	RE^d^	LE^e^	Log stereoacuity	Near	Distance
1	21	Female	−3.125	−2.875	1.600	−10.000	−2.000
2	21	Female	−7.750	−7.625	1.600	−4.000	0.000
3	21	Female	−1.875	−1.500	1.600	−8.000	−2.000
4	21	Female	−1.250	−0.250	1.600	2.000	0.000
5	21	Female	−0.625	−5.875	1.600	2.000	0.000
6	21	Female	−11.125	−11.125	1.600	−6.000	0.000
7	21	Female	−3.625	−3.750	1.600	−4.000	0.000
8	40	Female	−5.000	−5.000	1.600	−4.000	0.000
9	29	Male	−0.750	−0.750	1.600	−6.000	−2.000
10	25	Female	0.625	0.125	1.600	0.000	0.000
11	22	Female	−6.875	−5.000	1.600	−4.000	0.000
12	26	Male	−3.000	−3.000	1.600	−4.000	0.000
13	21	Female	−7.125	−7.875	1.600	−4.000	0.000
14	21	Female	−7.250	−5.750	1.600	−10.000	−2.000
15	21	Female	−3.250	−2.875	1.600	−8.000	0.000
16	33	Male	0.000	0.000	1.600	0.000	0.000
17	33	Male	−3.500	−2.625	1.600	−4.000	0.000
18	32	Female	0.000	0.250	1.600	−2.000	0.000
19	21	Female	0.000	−0.125	1.600	6.000	1.000
20	20	Female	−1.625	−0.750	1.600	−2.000	0.000

a D: diopter.

b PD: prism diopter.

c Ocular deviations with minus and plus signs indicate exodeviation and esodeviation, respectively.

d RE: right eye.

e LE: left eye.

### Subjective Symptom Scores Before and After Motion-Based VR Dichoptic Training

**Figure 4.**
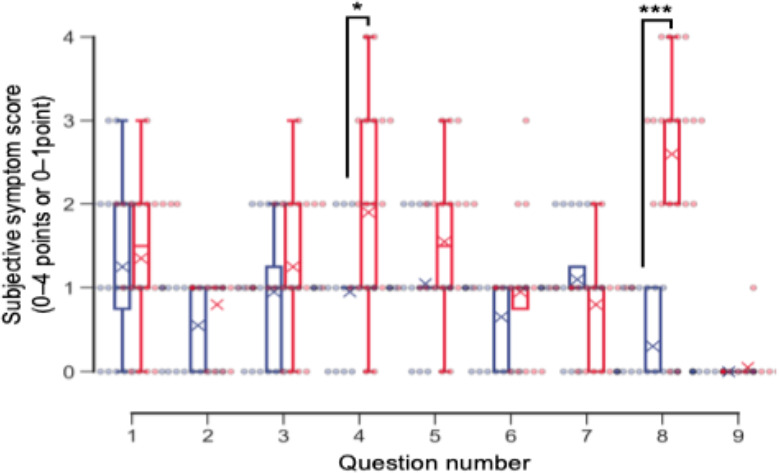
Subjective symptom scores before and after using the VR-based dichoptic training app. Box plots illustrating subjective symptom scores before (blue) and after (red) using the VR-based dichoptic training app in all participants (n=20). Dots represent individual data points. Significant increases were observed in back discomfort (Q4) and arm fatigue (Q8) after the task. *P=.03, Wilcoxon signed rank test with Holm correction. ***P<.001, Wilcoxon signed rank test with Holm correction. VR: virtual reality*.*

Subjective symptoms were evaluated using a 9-item questionnaire (Figure 3) [26-28]. Table 2 and Figure 4 show the subjective symptom scores before and after using the motion-based VR dichoptic training app.

No significant differences were observed in subjective eye symptoms (Q1-Q3) before and after motion-based VR dichoptic training. In contrast, physical and mental discomfort (Q4; mean 0.95, SD 0.59 points vs 1.90, SD 1.18 points; n=20; *P*=.03) and arm fatigue (Q8; mean 0.30, SD 0.46 points vs 2.60, SD 1.11 points; n=20; *P*<.001) were significantly greater following the task. None of the participants experienced VIMS (Q9; mean 0.00, SD 0.00 points vs 0.05, SD 0.22 points; n=20; *P*=.41) before or after the task. Of the 20 participants, 1 (5%) participant reported experiencing VIMS after the motion-based VR dichoptic training app.

**Table 2.** Subjective symptoms before and after using the motion-based VR^a^ dichoptic training app.

Subjective symptom questions	Motion-based VR dichoptic training app (n=20)		*P* value^b^
	Before, mean (SD)	After, mean (SD)	
Q1. How tired are your eyes? (0–4 points)	1.25 (0.94)	1.35 (0.85)	.41
Q2. How clear is your vision? (0–4 points)	0.55 (0.50)	0.80 (0.40)	.24
Q3. How does your eye feel? (0–4 points)	0.95 (0.74)	1.25 (0.83)	.08
Q4. How tired is your back? (0–4 points)	0.95 (0.59)	1.90 (1.18)	.03
Q5. How tired is your neck? (0–4 points)	1.05 (0.59)	1.55 (0.86)	.09
Q6. How severe is your headache? (0–4 points)	0.65 (0.48)	0.95 (0.74)	.19
Q7. How sleepy do you feel? (0–4 points)	1.10 (0.62)	0.80 (0.60)	.08
Q8. How tired are your arm or wrist? (0–4 points)	0.30 (0.46)	2.60 (1.11)	<.001
Q9. Do you feel visually induced motion sickness? (0=no or 1=yes point)	0.00 (0.00)	0.05 (0.22)	.41

a VR: virtual reality.

b The *P* values were adjusted using the Holm correction method.

## Discussion

### Principal Results

Pediatric amblyopia is a leading cause of childhood visual impairment, and while current treatments, such as corrective glasses and patch therapy, can improve visual acuity, they often exhibit low treatment adherence, cause stress for pediatric patients, and fail to fully address hand-eye coordination deficits. In this prospective intervention study, we investigated the potential ocular adverse effects of the motion-based VR dichoptic training app, designed for pediatric amblyopia treatment with enhanced hand-eye coordination. To ensure safety before implementation in pediatric patients, we first evaluated its effects in healthy adults, focusing on potential ocular adverse effects. The results showed no significant ocular symptoms after a 30-minute motion-based VR-dichoptic training, including eye fatigue, blurred vision, eye dryness, and VIMS. These findings suggest that the motion-based VR dichoptic training app is a low-risk intervention with minimal ocular side effects in healthy adults. Further studies are required to evaluate its safety, feasibility, and effectiveness in pediatric patients with amblyopia.

### Ocular Adverse Effects

This study found that visual fatigue did not significantly differ before and after using the motion-based VR dichoptic training app. Hirota et al [27] reported that 30-minute VR-HMD sessions induced a similar level of visual fatigue as conventional 2D displays. This discrepancy between their findings and ours may be attributed to differences in VR headset technology. The VR-HMD used in this study (VIVE Focus) differs from that of Hirota et al [27] (PlayStation VR). VR headsets released around 2016, such as the PlayStation VR, typically featured approximately 2K resolution, 100° field of view, and 90 Hz refresh rate. In contrast, modern VR headsets offer approximately 4K resolution, a 200° field of view, and a 120 Hz refresh rate. Additionally, advancements in motion sensor technology have enabled more precise synchronization between the avatar’s movements in the virtual environment and the user’s hand controller movements in real space. Moreover, the unique characteristics of the VR content itself may have contributed to the lack of significant visual fatigue in this study. These findings suggest that our motion-based VR dichoptic training app offers an active, gamified VR-based experience with minimal visual fatigue in healthy adults.

In this study, the absence of complaints regarding eye dryness following the use of the motion-based VR dichoptic training app likely reflects the nature of the training task. Video games often encourage continuous visual fixation on the screen, reducing blink frequency and leading to eye dryness [30]. However, in this study, the participants engaged in 5 Kendama wazas (techniques)—“Ozara (big dish),” “Kozara (small dish),” “Moshikame,” “Orbit,” and “Side spike.” When a waza was successfully completed, participants could continue playing, whereas unsuccessful attempts required them to look away and interact with a touch panel to reset the task. We believe these intermittent visual breaks helped prevent excessive tear evaporation and subsequent eye dryness.

This study also demonstrated that VIMS was not induced by 30 minutes of motion-based VR dichoptic training app use. VIMS occurs due to a discrepancy between visual and somatic sensations, which can be influenced by 3 factors: hardware, content, and human physiology [31]. In terms of hardware, VIVE Focus operates at a high sampling rate, reducing discomfort associated with image perception. Regarding content, the binocular disparity was minimized by adjusting the relative positions of the “tama (ball)” and the “sara (dish)” in the VR space. Jackson and Bedell [32] reported that correcting vertical heterophoria can reduce VIMS symptoms. In this study, adjusting both horizontal and vertical heterophoria to zero in the VR space may have contributed to the suppression of VIMS and enhanced visual comfort. This correction ensures that participants with varying degrees of heterophoria can experience more stable binocular alignment, potentially reducing discomfort and improving the overall usability of the VR app. Additionally, the nature of the movements in our VR task—controlled and primarily upper-body focused—may have contributed to minimizing VIMS risk by reducing the sensory mismatch between visual and vestibular inputs. Finally, regarding human factors, differences between adults and children may influence the incidence of VIMS when pediatric patients with amblyopia use our motion-based VR dichoptic training app in the future. However, our findings indicate that, at least in terms of hardware and content, the motion-based VR dichoptic training app has been designed to minimize conditions that induce VIMS.

### Physical and Mental Adverse Effects

Notably, some participants reported physical discomfort, primarily back discomfort, and arm fatigue, after the 30-minute motion-based VR dichoptic training session. These complaints are likely due to the weight of the VR-HMD and controller. Previous studies on VR-HMD-based amblyopia treatment have not reported physical adverse effects [16-18]. Since this study involved young adults with more developed musculature than children, pediatric patients using VR-HMDs may experience even greater back strain depending on their body weight. This concern is particularly relevant as children undergoing treatment would likely wear these devices regularly, potentially multiple times per day as part of their prescribed therapeutic regimen. Prolonged use of heavy VR-HMDs during critical growth periods could impact both physical and mental development [33,34]. Additionally, close monitoring of task performance is crucial when children use commercially available VR-HMDs. The observed arm fatigue likely stemmed from the participant’s unfamiliarity with the task and the extended session duration (30 minutes). A systematic review of VR interventions for children with cerebral palsy has reported effective results with session durations of 20‐30 minutes [35]. While the optimal duration for VR-based dichoptic training remains to be determined, these findings suggest that limiting session length may help balance efficacy with user comfort. Therefore, future studies involving pediatric patients should consider the use of lighter VR-HMDs and controllers, as well as shorter session durations, to optimize usability and minimize physical strain.

### Limitations

Despite the valuable insights gained from this study, some limitations should be acknowledged. First, this study assessed eye fatigue and VIMS for 30 minutes of motion-based VR dichoptic training app use in healthy adult participants, which aligns with the previous studies [13,16,18]. However, the effects of the app on eye fatigue and VIMS should be further evaluated in adult patients with amblyopia and in case of good tolerance, in pediatric patients with amblyopia. Second, Q8 of the subjective symptom questionnaire was originally designed to assess VR session–induced arm fatigue, while Q9 was intended to measure VIMS. These questions have not been assessed for validity and reliability, as no standardized questionnaire currently exists for assessing VIMS during VR use involving physical activity. Future research should incorporate a validated questionnaire to evaluate VIMS more accurately in this context. Third, adapting the use of VR-HMDs for pediatric patients with amblyopia requires ensuring a proper fit, accommodating a range of interpupillary distances, and minimizing the VR-HMD weight. This is particularly important because most commercially available VR-HMDs are optimized for adult interpupillary distance ranges, which may not be suitable for younger children and could affect alignment and comfort. While this study demonstrated the safety of the motion-based VR dichoptic training app in healthy adult participants, further refinements are necessary before its implementation in pediatric patients. Moving forward, we intend to conduct further investigations in adult patients who have previously undergone amblyopia treatment but are currently experiencing declining visual acuity in one eye. This approach would allow us to refine and validate the treatment protocol without requiring additional hardware development.

### Conclusions

Our 30-minute motion-based VR dichoptic training app session resulted in minimal visual discomfort, including minimal eye fatigue, blurred vision, eye dryness, and VIMS. These results provide preliminary evidence that daily amblyopia training using motion-based VR dichoptic training apps may minimize adverse ocular effects. Future studies in adults and children with amblyopia are needed to assess the tolerability and efficacy of the motion-based VR dichoptic training app for the treatment of patients with amblyopia.

## Abbreviations

HMD: head-mounted display
VIMS: visually induced motion sickness
VR: virtual reality

## References

[1] Attebo K, Mitchell P, Cumming R, Smith W, Jolly N, Sparkes R (1998). Prevalence and causes of amblyopia in an adult population. Ophthalmology.

[2] Faghihi M, Hashemi H, Nabovati P (2017). The prevalence of amblyopia and its determinants in a population-based study. Strabismus.

[3] Wu C, Hunter DG (2006). Amblyopia: diagnostic and therapeutic options. Am J Ophthalmol.

[4] Liang YB, Friedman DS, Wong TY (2008). Prevalence and causes of low vision and blindness in a rural chinese adult population: the Handan Eye Study. Ophthalmology.

[5] de Zárate BR, Tejedor J (2007). Current concepts in the management of amblyopia. Clin Ophthalmol.

[6] Tan JHY, Thompson JR, Gottlob I (2003). Differences in the management of amblyopia between European countries. Br J Ophthalmol.

[7] Moseley MJ, Fielder AR, Irwin M, Jones HS, Auld RJ (1997). Effectiveness of occlusion therapy in ametropic amblyopia: a pilot study. Br J Ophthalmol.

[8] Scheiman MM, Hertle RW, Beck RW (2005). Randomized trial of treatment of amblyopia in children aged 7 to 17 years. Arch Ophthalmol.

[9] Wallace MP, Stewart CE, Moseley MJ (2013). Compliance with occlusion therapy for childhood amblyopia. Invest Ophthalmol Vis Sci.

[10] Searle A, Norman P, Harrad R, Vedhara K (2002). Psychosocial and clinical determinants of compliance with occlusion therapy for amblyopic children. Eye (Lond).

[11] Birch EE (2013). Amblyopia and binocular vision. Prog Retin Eye Res.

[12] Hess RF, Mansouri B, Thompson B (2010). A new binocular approach to the treatment of amblyopia in adults well beyond the critical period of visual development. Restor Neurol Neurosci.

[13] Emmanouil B, Spaho J, Chatzea M, Gleni A, Plainis S (2023). Dichoptic game training in strabismic amblyopia improves the visual evoked response. Cureus.

[14] Vedamurthy I, Nahum M, Huang SJ (2015). A dichoptic custom-made action video game as a treatment for adult amblyopia. Vision Res.

[15] Mon-Williams M, Wann JP, Rushton S (1993). Binocular vision in a virtual world: visual deficits following the wearing of a head-mounted display. Ophthalmic Physiol Opt.

[16] Žiak P, Holm A, Halička J, Mojžiš P, Piñero DP (2017). Amblyopia treatment of adults with dichoptic training using the virtual reality oculus rift head mounted display: preliminary results. BMC Ophthalmol.

[17] Elhusseiny AM, Bishop K, Staffa SJ, Zurakowski D, Hunter DG, Mantagos IS (2021). Virtual reality prototype for binocular therapy in older children and adults with amblyopia. J AAPOS.

[18] Xiao S, Angjeli E, Wu HC (2022). Randomized controlled trial of a dichoptic digital therapeutic for amblyopia. Ophthalmology.

[19] Rajavi Z, Soltani A, Vakili A (2021). Virtual reality game playing in amblyopia therapy: a randomized clinical trial. J Pediatr Ophthalmol Strabismus.

[20] Meqdad Y, El-Basty M, Awadein A, Gouda J, Hassanein D (2024). Randomized controlled trial of patching versus dichoptic stimulation using virtual reality for amblyopia therapy. Curr Eye Res.

[21] Suttle CM, Melmoth DR, Finlay AL, Sloper JJ, Grant S (2011). Eye-hand coordination skills in children with and without amblyopia. Invest Ophthalmol Vis Sci.

[22] Grant S, Suttle C, Melmoth DR, Conway ML, Sloper JJ (2014). Age- and stereovision-dependent eye-hand coordination deficits in children with amblyopia and abnormal binocularity. Invest Ophthalmol Vis Sci.

[23] Nakazawa T, Okubo Y, Suwazono Y (2002). Association between duration of daily VDT use and subjective symptoms. Am J Ind Med.

[24] Sheedy J, Bergstrom N (2002). Performance and comfort on near-eye computer displays. Optom Vis Sci.

[25] Hoffman DM, Girshick AR, Akeley K, Banks MS (2008). Vergence-accommodation conflicts hinder visual performance and cause visual fatigue. J Vis.

[26] Hirota M, Yada K, Morimoto T (2020). Objective evaluation of visual fatigue in patients with intermittent exotropia. PLoS ONE.

[27] Hirota M, Kanda H, Endo T (2019). Comparison of visual fatigue caused by head-mounted display for virtual reality and two-dimensional display using objective and subjective evaluation. Ergonomics.

[28] Hirota M, Morimoto T, Kanda H (2018). Objective evaluation of visual fatigue using binocular fusion maintenance. Transl Vis Sci Technol.

[29] Leal-Vega L (2024). NEIVATECH pilot study: immersive virtual reality training in older amblyopic children with non-compliance or non-response to patching. Sci Rep.

[30] Tsubota K, Nakamori K (1993). Dry eyes and video display terminals. N Engl J Med.

[31] Chang E, Kim HT, Yoo B (2020). Virtual reality sickness: a review of causes and measurements. Int J Hum-Comput Interact.

[32] Jackson DN, Bedell HE (2012). Vertical heterophoria and susceptibility to visually induced motion sickness. Strabismus.

[33] Briggs AM, Smith AJ, Straker LM, Bragge P (2009). Thoracic spine pain in the general population: prevalence, incidence and associated factors in children, adolescents and adults. A systematic review. BMC Musculoskelet Disord.

[34] Rodríguez-Oviedo P, Ruano-Ravina A, Pérez-Ríos M (2012). School children’s backpacks, back pain and back pathologies. Arch Dis Child.

[35] Ghai S, Ghai I (2019). Virtual reality enhances gait in cerebral palsy: a training dose-response meta-analysis. Front Neurol.

